# Preexisting Neuropsychiatric Conditions and Associated Risk of Severe COVID-19 Infection and Other Acute Respiratory Infections

**DOI:** 10.1001/jamapsychiatry.2022.3614

**Published:** 2022-11-09

**Authors:** Tom Alan Ranger, Ash Kieran Clift, Martina Patone, Carol A. C. Coupland, Robert Hatch, Karen Thomas, Peter Watkinson, Julia Hippisley-Cox

**Affiliations:** 1 Nuffield Department of Primary Care Health Sciences, University of Oxford, Oxford, United Kingdom; 2 Cancer Research UK Oxford Centre, University of Oxford, Oxford, United Kingdom; 3 Division of Primary Care, School of Medicine, University of Nottingham, Nottingham, United Kingdom; 4 Nuffield Department of Clinical Neurosciences, University of Oxford, Oxford Biomedical Research Centre, Oxford University Hospitals NHS Trust, Oxford, United Kingdom; 5 Intensive Care National Audit and Research Centre, London, Oxford, United Kingdom

## Abstract

**Importance:**

Evidence indicates that preexisting neuropsychiatric conditions confer increased risks of severe outcomes from COVID-19 infection. It is unclear how this increased risk compares with risks associated with other severe acute respiratory infections (SARIs).

**Objective:**

To determine whether preexisting diagnosis of and/or treatment for a neuropsychiatric condition is associated with severe outcomes from COVID-19 infection and other SARIs and whether any observed association is similar between the 2 outcomes.

**Design, Setting, and Participants:**

Prepandemic (2015-2020) and contemporary (2020-2021) longitudinal cohorts were derived from the QResearch database of English primary care records. Adjusted hazard ratios (HRs) with 99% CIs were estimated in April 2022 using flexible parametric survival models clustered by primary care clinic. This study included a population-based sample, including all adults in the database who had been registered with a primary care clinic for at least 1 year. Analysis of routinely collected primary care electronic medical records was performed.

**Exposures:**

Diagnosis of and/or medication for anxiety, mood, or psychotic disorders and diagnosis of dementia, depression, schizophrenia, or bipolar disorder.

**Main Outcomes and Measures:**

COVID-19–related mortality, or hospital or intensive care unit admission; SARI-related mortality, or hospital or intensive care unit admission.

**Results:**

The prepandemic cohort comprised 11 134 789 adults (223 569 SARI cases [2.0%]) with a median (IQR) age of 42 (29-58) years, of which 5 644 525 (50.7%) were female. The contemporary cohort comprised 8 388 956 adults (58 203 severe COVID-19 cases [0.7%]) with a median (IQR) age of 48 (34-63) years, of which 4 207 192 were male (50.2%). Diagnosis and/or treatment for neuropsychiatric conditions other than dementia was associated with an increased likelihood of a severe outcome from SARI (anxiety diagnosis: HR, 1.16; 99% CI, 1.13-1.18; psychotic disorder diagnosis and treatment: HR, 2.56; 99% CI, 2.40-2.72) and COVID-19 (anxiety diagnosis: HR, 1.16; 99% CI, 1.12-1.20; psychotic disorder treatment: HR, 2.37; 99% CI, 2.20-2.55). The effect estimate for severe outcome with dementia was higher for those with COVID-19 than SARI (HR, 2.85; 99% CI, 2.71-3.00 vs HR, 2.13; 99% CI, 2.07-2.19).

**Conclusions and Relevance:**

In this longitudinal cohort study, UK patients with preexisting neuropsychiatric conditions and treatments were associated with similarly increased risks of severe outcome from COVID-19 infection and SARIs, except for dementia.

## Key Points

**Question:**

Is there an association between having a diagnosis of a neuropsychiatric condition and severe outcome from COVID-19 infection, and if so, is it also observed in other severe acute respiratory infections (SARIs)?

**Findings:**

In this longitudinal cohort study of the electronic medical records from more than 11 million people, both a preexisting diagnosis of a neuropsychiatric condition and having a prescription for a related pharmacological treatment were associated with a significantly increased risk of severe outcomes from both COVID-19 infection and other SARIs, mainly to similar extents.

**Meaning:**

These findings suggest the importance of recognizing the association of several neuropsychiatric illnesses with risk of developing a broad range of respiratory infections, not just COVID-19 infection.

## Teaser

This cohort study investigates the potential association between a previous diagnosis of a neuropsychiatric condition and severe outcome from COVID-19 infection and other severe acute respiratory infections.

## Introduction

The COVID-19 pandemic has caused at least 5 million deaths and continues to exert significant pressures on health care systems globally.^1^ Despite successful vaccination strategies in many countries, it remains important to be aware of conditions that may predispose to worse outcomes from SARS-CoV-2 infection so that at-risk populations can be identified for the purposes of public health strategy.

Recent evidence indicates that the risk of developing an incident neuropsychiatric condition after severe COVID-19 infection and a severe acute respiratory infection (SARI) is similar.^2^ Moreover, 3 systematic reviews with meta-analysis^3,4,5^ and a subsequent major cohort study have^6^ reported that people with preexisting neuropsychiatric conditions are at higher risk of COVID-19 mortality than people without such a diagnosis. These reports align with prepandemic data suggesting that severe mental illness,^7^ schizophrenia,^8^ and depression^9^ are associated with increased risks of developing SARIs. It remains unclear, however, whether the increased risk of developing severe disease associated with these conditions differs between COVID-19 infection and other SARIs.

Before the COVID-19 pandemic, results of studies^10,11,12^ suggested that some drug classes typically prescribed for these neuropsychiatric conditions have also been associated with increased risk of more severe respiratory infection. Evidence for some drug classes, however (eg, antidepressants), is conflicting.^13,14^ There is a need for greater understanding of the associations between medications typically prescribed for psychiatric conditions and severe respiratory infection.

As COVID-19 becomes endemic, it is critical to understand which medical conditions and treatments may predispose to more severe disease. Furthermore, understanding whether neuropsychiatric conditions contribute to a generalized increase in risk for acute respiratory illness or whether this risk is disease specific is important for ongoing management of individuals and health systems. This study aimed, therefore, to evaluate the associations of preexisting neuropsychiatric conditions and treatments with COVID-19 outcomes compared to those with SARI.

## Methods

This longitudinal cohort study was conducted in accordance with our prespecified protocol^15^; deviations from the protocol are described in the protocol deviation section of the methods. The study was approved by the QResearch Scientific Committee, which has ethical approval capabilities from the East Midlands–Derby research ethics committee, including a project-specific patient waiver for patient consent forms due to data protections (including deidentification) implemented as part of the agreement. This study followed the Strengthening the Reporting of Observational Studies in Epidemiology (STROBE) reporting guidelines.^16^

We used the QResearch database of English primary care records, version 45 (EMIS Health), which has individual-level linkage to Hospital Episode Statistics (HES), Public Health England’s second-generation surveillance system database regarding SARS-CoV-2 testing, the Intensive Care National Audit and Research Centre (ICNARC) database, and the Office for National Statistics’ national mortality register. All records in the database meeting inclusion criteria were used. Patients self-reported their race or ethnicity as one of Asian, Black, White, or other if they did not identify with one of the previous 3 categories. Completion of this variable is not mandatory in primary care clinics; therefore, in some cases, it is missing.

We extracted 2 temporally distinct longitudinal cohorts: a prepandemic cohort comprising all adults (18 years and older) entering from January 24, 2015, until January 23, 2020 (the day before first recorded COVID-19 case in the UK), and a contemporary cohort comprising all adults entering from January 24, 2020, until the date of data extraction (May 31, 2021). The index date in the prepandemic cohort was the latest of the following: (1) January 24, 2015; (2) December 31 in the year that patients turned 18 years; or (3) 1 year after registration with a participating practice if registration was after January 24, 2014. The index date for the contemporary cohort was January 24, 2020 (first recorded SARS-CoV-2 infection in the UK), with participants younger than 18 years or not registered with a participating practice for at least 1 year on that date excluded. Patient follow-up time was from index date until the first record of any outcome (COVID-19– or SARI-related hospital or ICU admission, or death) or censoring: the earliest of date of deregistration from an EMIS practice or study end (January 23, 2020, for the prepandemic cohort and May 31, 2021, for the contemporary cohort).

### Exposures

Detailed definitions of exposures are reported in full in the study protocol.^15^ Briefly, we used SNOMED/Read Codes in the primary care records, and *International Statistical Classification of Diseases and Related Health Problems, Tenth Revision (ICD-10) *codes in the HES records to identify individuals with selected neuropsychiatric conditions. We used primary care records to identify prescriptions of relevant medications based on the British National Formulary.^17^ We grouped similar neuropsychiatric conditions and their respective pharmacological treatments under the following categories: anxiety, mood, and psychotic disorders and hypnotic/anxiolytic, antidepressant, and antipsychotic medications. The primary care code lists used to create these groups are available on the QResearch website.^18^ In addition, we investigated diagnosis only (ie, irrespective of treatment) of the following conditions: depression, dementia, schizophrenia, and bipolar disorder. Finally, we investigated any antidepressant use irrespective of concurrent diagnosis. Patients were considered exposed if they had a diagnosis of a neuropsychiatric condition at any time before the index date, and/or at least 2 prescriptions for neuropsychiatric medications in the 6 months before baseline. Patients with a first-ever diagnosis of a neuropsychiatric condition or 2 or more prescriptions for related medications within 6 months during the follow-up time were excluded from analysis for the relevant condition to avoid immortal time bias.^19^

### Outcomes

Using these cohorts, we estimated associations between prior neuropsychiatric conditions and relevant medication use and subsequent severe COVID-19 or SARI-related illness (in the contemporary and prepandemic cohorts, respectively). A severe outcome was defined as hospitalization, intensive care unit (ICU) admission, or death related to COVID-19 infection or SARI. Hospital admissions of any duration were identified using linked HES data and were considered COVID-19 related if the person had a positive SARS-CoV-2 test result within 14 days before or during admission or if they had *ICD-10* code U07.01 in their HES record (indicating confirmed COVID-19 infection). For ICU admissions, we used ICNARC’s data set of COVID-19–related admissions. Death from any cause within 28 days of a positive COVID-19 test or where COVID-19 was listed as a cause of death on the death certificate was considered COVID-19 related. Hospital admissions were considered SARI related if any *ICD-10* code from J09-J22 occurred in the HES record. Deaths where the same *ICD-10* codes were listed as a cause of death on the death certificate were considered SARI related. SARI-related ICU admission was determined based on corresponding RE, or respiratory, diagnosis codes used internally by ICNARC.^15^ For both conditions, a composite outcome of severe COVID-19 infection or SARI was created of the first recorded of hospitalization, ICU admission, or death.

### Statistical Analyses

Flexible parametric survival models with clustering by general primary care practice were used to calculate hazard ratios (HRs) and 99% CIs, to assess whether prior neuropsychiatric diagnosis or treatment was associated with the risk of severe COVID-19 infection or SARI. All final analysis models were adjusted for demographic and clinical factors identified from clinical/epidemiologic understanding, as summarized in the directed acyclic graphs in the protocol.^15^

Missing data were observed for race and ethnicity, smoking status, alcohol status, body mass index, and Townsend deprivation score quintile; multiple imputation with chained equations was used to replace these under the missing at random assumption.^20^ Imputation models included all covariates and end points. Five imputations were generated, with model coefficients and SEs pooled in accordance with Rubin rules.^21^ *P* values were 2-sided, and results were considered statistically significant if the 99% CI did not include 1. All analyses were conducted in Stata, version 17 (StataCorp).

#### Protocol Deviation

In some instances, we deviated from the original study protocol. Additional exposures were analyzed beyond those listed in the protocol based on clinical relevance; these included the following: bipolar disorder (*ICD-10* codes F30-31 and corresponding Read Codes), depression (*ICD-10* F06.3, F32-F33, F34.1, F41.2, F44.8, F92.0, and corresponding Read Codes), schizophrenia (*ICD-10* F06.2, F20, F23.0-F23.2, and corresponding Read Codes), and antidepressant use (tricyclic and related antidepressant drugs, monoamine-oxidase inhibitors, selective serotonin reuptake inhibitors [SSRIs], other antidepressant drugs). Bipolar disorder is included in the broader category of mood disorders, schizophrenia is included in psychotic disorders, and antidepressant use is considered the treatment for mood disorders. Nevertheless, bipolar disorder, schizophrenia, and antidepressant use were highlighted for their particular clinical importance, and we have been careful not to overinterpret the results beyond those of the broader categories.

A frailty term for general practice was proposed for the proportional hazards models, but the use of Royston-Parmar models (Stata package stpm2) precluded this. Instead, we calculated SEs adjusted for clustering at the general practice level.

#### Sensitivity Analysis

A sensitivity analysis was added to investigate the potential confounding effect of COVID-19 vaccination. In this analysis, the end day in the contemporary cohort was changed to December 7, 2020, the day before the first COVID-19 vaccination was administered in the UK. Pilot analysis showed similar findings when hospitalization, ICU admission, and death were investigated separately; therefore, only the combined variable was used in the main analysis. An analysis of COVID-19– and SARI-specific mortality is included in eTables 1, 2, and 3 in the Supplement.

## Results

There were 11 134 789 individuals in the prepandemic cohort (5 644 525 female [50.7%]; 5 490 264 male [49.3%]) and 8 388 956 people in the contemporary cohort (1 181 764 female [49.8%]; 4 207 192 male [50.2%]). The 2 cohorts were broadly similar in terms of lifestyle and demographic factors (Table 1), comorbidities, and prescribed medications (Table 2). The contemporary cohort, however, was older (median [IQR] age, prepandemic cohort, 42 [29-58] years vs contemporary cohort, 48 [34-63] years). The following race and ethnicity categories were included in the prepandemic cohort: 986 264 Asian (8.9%), 381 244 Black (3.4%), 7 094 605 White (63.7%), 2 270 543 missing (20.4%), and 402 133 other (3.6%). The following race and ethnicity categories were included in the contemporary cohort: 735 644 Asian (8.8%), 285 126 Black (3.4%), 5 389 025 White (64.2%), 1 690 035 missing (20.1%), and 289 126 other (3.4%). The prevalence of diagnosis only of anxiety at the index date was higher in the contemporary cohort than the prepandemic cohort (1 013 565 [12.1%] vs 999 125 [9.0%]) (Table 1). Prevalence of diagnosis of other neuropsychiatric conditions appeared broadly similar between the 2 cohorts. Median (IQR) follow-up was 1275 (496-1825) days in the prepandemic cohort and 412 (375-493) days in the contemporary cohort. The number of patients with a SARI outcome in the prepandemic cohort during follow-up was 223 569 (2.0%), and the number of patients with a severe COVID-19 outcome in the contemporary cohort during follow-up was 58 203 (0.7%) (Table 1). There were 21 649 COVID-19–related deaths in the contemporary cohort study period; of these, 932 were within 28 days of a positive test result, 5183 had COVID-19 listed on the death certificate, and 15 534 had both. The numbers of patients with a neuropsychiatric condition who developed SARI or COVID-19 illness are shown in Table 3 for each analysis.

**Table 1.** Summary Information on Demographic and Lifestyle Factors and Exposures for the Prepandemic Severe Acute Respiratory Infection (SARI) and Contemporary COVID-19 Infection Cohorts at the Index Date and Outcomes During Follow-up

Characteristic	No. (%)	
	Prepandemic cohort: SARI (2015-2020)	Contemporary cohort: COVID-19 (2020-2021)
Total No. of patients	11 134 789	8 388 956
Follow-up time, median (IQR), d	1275 (496- 1825)	412 (375- 493)
Age, median (IQR), y	42 (29- 58)	48 (34- 63)
Sex		
Female	5 644 525 (50.7)	4 181 764 (49.8)
Male	5 490 264 (49.3)	4 207 192 (50.2)
BMI^a^		
Median (IQR)	26 (23- 30)	26 (23- 30)
Missing	1 773 555 (15.9)	1 212 590 (14.5)
Severe outcome during follow-up^b^	SARI	COVID-19
First recorded	223 569 (2.0)	58 203 (0.7)
Hospital admission (excluding ICU)	196 232 (1.8)	56 592 (0.7)
ICU admission	17 025 (0.2)	6410 (0.1)
Death	82 557 (0.7)	21 649 (0.3)
Anxiety^c^		
Diagnosis only	999 125 (9.0)	1 013 565 (12.1)
Diagnosis and treatment	62 194 (0.6)	50 899 (0.6)
Treatment only	114 993 (1.0)	74 909 (0.9)
Excluded	459 682 (28.1)	113 825 (9.1)
Mood disorder^b^		
Diagnosis only	585 993 (5.3)	514 007 (6.1)
Diagnosis and treatment	366 144 (3.3)	348 768 (4.2)
Treatment only	603 914 (5.4)	576 955 (6.9)
Excluded	782 124 (33.5)	207 730 (12.6)
Psychotic disorder^b^		
Diagnosis only	30 262 (0.3)	23 194 (0.3)
Diagnosis and treatment	34 519 (0.3)	26 866 (0.3)
Treatment only	82 698 (0.7)	67 288 (0.8)
Excluded	7425 (8.0)	22 190 (15.9)
Dementia^b^	132 622 (1.2)	101 777 (1.2)
Excluded	80 406 (37.7)	15 856 (13.5)
Schizophrenia^b^	55 374 (0.5)	42 061 (0.5)
Excluded	4973 (8.2)	970 (2.3)
Depression^b^	921 948 (8.3)	825 732 (9.8)
Excluded	140 725 (13.2)	5445 (0.7)
Bipolar^b^	46 379 (0.4)	38 491 (0.5)
Excluded	6811 (12.8)	4861 (11.2)
Antidepressant treatment^b^	1 017 859 (9.1)	928 341 (11.1)
Excluded	591 281 (36.8)	198 161 (17.6)
Region of England		
East Midlands	307 004 (2.8)	205 770 (2.5)
East of England	419 157 (3.8)	325 733 (3.9)
London	2 905 806 (26.1)	2 035 931 (24.3)
Northeast	269 553 (2.4)	206 092 (2.5)
Northwest	2 002 153 (18.0)	1 578 863 (18.8)
South Central	1 415 670 (12.7)	1 089 524 (13.0)
Southeast	1 166 717 (10.5)	915 109 (10.9)
Southwest	1 131 089 (10.2)	859 409 (10.2)
West Midlands	106 9962 (9.6)	845 864 (10.1)
Yorkshire and Humber	44 7678 (4.0)	32 6661 (3.9)
Townsend quintile		
1 (Least deprived)	2 583 677 (23.2)	2 083 583 (24.8)
2	2 332 640 (20.9)	1 834 284 (21.9)
3	2 159 320 (19.4)	1 637 994 (19.5)
4	2 012 583 (18.1)	1 452 158 (17.3)
5 (Most deprived)	2 002 838 (18.0)	1 342 583 (16.0)
Missing	43 731 (0.4)	38 354 (0.5)
Race and ethnicity		
Asian	986 264 (8.9)	735 644 (8.8)
Black	381 244 (3.4)	285 126 (3.4)
White	7 094 605 (63.7)	5 389 025 (64.2)
Missing	2 270 543 (20.4)	1 690 035 (20.1)
Other^d^	402 133 (3.6)	289 126 (3.4)
Smoking status		
Nonsmoker	6 393 932 (57.4)	4 840 921 (57.7)
Ex-smoker	2 325 940 (20.9)	1 827 288 (21.8)
Light	1 506 588 (13.5)	1 050 847 (12.5)
Moderate	290 564 (2.6)	229 416 (2.7)
Heavy	134 483 (1.2)	106 090 (1.3)
Missing	483 282 (4.3)	334 394 (4.0)
Alcohol consumption, U/d		
Nondrinker	5 703 941 (51.2)	4 382 572 (52.2)
Trivial <1	1 631 593 (14.7)	1 249 772 (14.9)
Light 1-2	825 218 (7.4)	635 506 (7.6)
Moderate 3-6	614 568 (5.5)	487 122 (5.8)
Heavy 7-9	49 275 (0.4)	38 936 (0.5)
Very heavy >9	53 900 (0.5)	35 720 (0.4)
Missing	2 256 294 (20.3)	1 559 328 (18.6)

Abbreviations: BMI, body mass index; ICU, intensive care unit.

^a^ Calculated as weight in kilograms divided by height in meters squared.

^b^ Indicates total numbers recorded for each event during the relevant study period; therefore, a person may appear in more than 1 row if they, for example, are hospitalized and subsequently die. The row first recorded is the earliest record of any event; therefore, people will only appear once.

^c^ Diagnoses defined as any record before baseline; treatment defined as 2 or more prescriptions in the 6 months before baseline; percentages of cases are of the total cohort; percentages of the number excluded are of total number diagnosed.

^d^ Other race and ethnicity includes people who did not identify with any of the previous 3 options.

**Table 2.** Summary Information on Comorbidites and Medications for the Prepandemic Severe Acute Respiratory Infection (SARI) and Contemporary COVID-19 Infection Cohorts at the Index Date

Comorbidities and medications	No. (%)	
	Prepandemic cohort: SARI (2015-2020)	Contemporary cohort: COVID-19 (2020-2021)
Total No. of patients	11 134 789	8 388 956
Neoplasms		
Gastrointestinal cancers	88 171 (0.8)	59 124 (0.7)
Urogenital cancers	134 940 (1.2)	106 473 (1.3)
Gynecological cancers	33 977 (0.3)	26 995 (0.3)
Breast cancer	133 106 (1.2)	109 137 (1.3)
Lung cancer	29 137 (0.3)	13 742 (0.2)
Hematological cancers	63 490 (0.6)	50 349 (0.6)
Pulmonary		
COPD	279 977 (2.5)	209 515 (2.5)
Asthma	1 473 736 (13.2)	1 147 463 (13.7)
Bronchiectasis	55 855 (0.5)	47 767 (0.6)
Rare pulmonary diseases	30 820 (0.3)	22 154 (0.3)
Circulatory		
Coronary heart disease	436 822 (3.9)	333 273 (4.0)
Hypertension	1 913 743 (17.2)	1 575 482 (18.8)
Congestive cardiac failure	160 651 (1.4)	119 851 (1.4)
Stroke	282 525 (2.5)	206 990 (2.5)
Peripheral vascular disease	98 505 (0.9)	69 596 (0.8)
Venous thromboembolism	211 999 (1.9)	171 474 (2.0)
Atrial fibrillation	308 170 (2.8)	237 653 (2.8)
Sickle cell anemia	8699 (0.1)	6976 (0.1)
Neurological		
Learning impediments	177 637 (1.6)	137 143 (1.6)
Severe head injury	58 509 (0.5)	46 947 (0.6)
Epilepsy	151 385 (1.4)	116 631 (1.4)
Multiple sclerosis	27 015 (0.2)	22 072 (0.3)
Skeletal		
Fracture	471 836 (4.2)	355 081 (4.2)
Osteoarthritis	1 200 356 (10.8)	997 346 (11.9)
Other conditions		
Rheumatoid diseases	98 820 (0.9)	81 073 (1.0)
Type 1 diabetes	64 244 (0.6)	48 433 (0.6)
Type 2 diabetes	753 013 (6.8)	623 174 (7.4)
Hypothyroidism	478 928 (4.3)	386 816 (4.6)
Renal complications	515 863 (4.6)	388 947 (4.6)
Chronic liver/pancreatic diseases	88 689 (0.8)	82 501 (1.0)
Bone marrow transplant	4466 (0.0)	3689 (0.0)
Medications^a^		
Steroids	815 596 (7.3)	289 510 (3.5)
Statins	1 693 840 (15.2)	1 314 861 (15.7)
NSAIDs	1 879 856 (16.9)	564 435 (6.7)
Aspirin	690 868 (6.2)	384 181 (4.6)
Estrogen	216 352 (1.9)	146 110 (1.7)
Progestogen	221 259 (2.0)	55 793 (0.7)
HRT combined	99 658 (0.9)	47 564 (0.6)
Anticonvulsants	673 395 (6.0)	373 428 (4.5)
Bisphosphonates	255 609 (2.3)	134 537 (1.6)
Leukotrienes	1 390 716 (12.5)	781 972 (9.3)
ACE inhibitors	1 108 285 (10.0)	770 690 (9.2)
Anticoagulants	358 004 (3.2)	274 708 (3.3)
Cytotoxins	33 888 (0.3)	18 641 (0.2)

Abbreviations: ACE, angiotensin-converting enzyme; COPD, chronic obstructive pulmonary disease; HRT, hormone replacement therapy; NSAID, nonsteroidal anti-inflammatory drug.

^a^ Medication use defined as any prescription during the study period.

**Table 3.** Cases^a^ and Regression Estimates From Univariate and Multivariable Royston-Parmar Flexible Parametric Survival Models, Clustered by Primary Care Clinic^b^

Neuropsychiatric condition	SARI (prepandemic cohort)			COVID-19 (contemporary cohort)		
	Cases, No. (%)	HR (99% CI)		Cases, No. (%)	HR (99% CI)	
		Univariate model	Maximally adjusted multivariable model		Univariate model	Maximally adjusted multivariable model
Anxiety disorder^c^						
No anxiety	171 092 (80.8)	1 [Reference]	1 [Reference]	46 611 (81.6)	1 [Reference]	1 [Reference]
Diagnosis only	24 276 (11.5)	1.32 (1.29-1.36)	1.16 (1.13-1.18)	7464 (13.1)	1.13 (1.09-1.17)	1.16 (1.12-1.20)
Diagnosis and treatment	5017 (2.4)	4.39 (4.19-4.59)	1.62 (1.55-1.69)	1003 (1.8)	3.06 (2.81-3.34)	1.53 (1.41-1.67)
Treatment only	11 286 (5.3)	5.70 (5.49-5.92)	1.67 (1.62-1.72)	2067 (3.6)	4.36 (4.08-4.67)	1.70 (1.59-1.81)
Mood disorder^d^						
No mood disorder	143 260 (69.7)	1 [Reference]	1 [Reference]	39 701 (69.9)	1 [Reference]	1 [Reference]
Diagnosis only	13 422 (6.5)	1.35 (1.31-1.40)	1.23 (1.20-1.27)	3714 (6.5)	1.23 (1.17-1.29)	1.20 (1.15-1.26)
Diagnosis and treatment	16 203 (7.9)	2.61 (2.52-2.71)	1.76 (1.71-1.80)	4730 (8.3)	2.28 (2.18-2.39)	1.63 (1.56-1.71)
Treatment only	32 561 (15.8)	3.23 (3.13-3.33)	1.66 (1.62-1.69)	8669 (15.3)	2.55 (2.45-2.65)	1.59 (1.53-1.65)
Psychotic disorder^e^						
No psychotic disorder	210 142 (95.7)	1 [Reference]	1 [Reference]	55 033 (95.4)	1 [Reference]	1 [Reference]
Diagnosis only	1245 (0.6)	2.44 (2.26-2.64)	1.90 (1.70-2.13)	356 (0.6)	2.45 (2.10-2.86)	1.89 (1.62-2.21)
Diagnosis and treatment	2191 (1.0)	3.40 (3.17-3.64)	2.56 (2.40-2.72)	604 (1.0)	3.43 (3.07-3.82)	2.19 (1.96-2.44)
Treatment only	6025 (2.7)	4.33 (4.13-4.55)	2.35 (2.26-2.45)	1669 (2.9)	3.85 (3.56-4.15)	2.37 (2.20-2.55)
Other diagnoses						
Dementia	191 001 (89.4)	1 [Reference]	1 [Reference]	50 621 (88.0)	1 [Reference]	1 [Reference]
	22 747 (10.6)	15.80 (15.22-16.40)	2.13 (2.07-2.19)	6900 (12.0)	13.45 (12.78-14.17)	2.85 (2.71-3.00)
Depression^f^	193 406 (87.7)	1 [Reference]	1 [Reference]	50 370 (86.6)	1 [Reference]	1 [Reference]
	27 242 (12.3)	1.53 (1.48-1.57)	1.31 (1.28-1.33)	7786 (13.4)	1.41 (1.36-1.46)	1.23 (1.19-1.27)
Bipolar disorder^g^	221 305 (99.0)	1 [Reference]	1 [Reference]	57 514 (98.9)	1 [Reference]	1 [Reference]
	2127 (1.0)	2.52 (2.37-2.69)	1.90 (1.77-2.05)	653 (1.1)	2.52 (2.26-2.81)	1.86 (1.68-2.07)
Schizophrenia^h^	220 391 (98.7)	1 [Reference]	1 [Reference]	57 331 (98.5)	1 [Reference]	1 [Reference]
	3012 (1.3)	2.90 (2.74-3.08)	2.21 (2.08-2.36)	858 (1.5)	3.08 (2.79-3.40)	2.05 (1.86-2.26)
Antidepressant treatment^i^	158 945 (76.2)	1 [Reference]	1 [Reference]	43 469 (76.4)	1 [Reference]	1 [Reference]
	49 600 (23.8)	2.89 (2.81-2.97)	1.64 (1.62-1.67)	13 427 (23.6)	2.41 (2.33-2.49)	1.57 (1.52-1.62)

Abbreviations: HR, hazard ratio; SARI, severe acute respiratory illness.

^a^ People with a diagnosis of or pharmacological treatment for a neuropsychiatric condition who develop SARI or COVID-19 infection.

^b^ Denominator for percentages is total number of people with the outcome (ie, SARI or COVID-19). Multivariable model adjusted for: age, sex, body mass index, ethnicity, Townsend index of socioeconomic deprivation, smoking and alcohol consumption, comorbidities, and other medications.

^c^ Anxiety diagnoses: *International Statistical Classification of Diseases and Related Health Problems, Tenth Revision (ICD-10)* F40-48 and corresponding Read Codes.

^d^ Mood disorder: *ICD-10* F30-39 and corresponding Read Codes.

^e^ Psychotic disorders: *ICD-10* F00-03, G30, G31.0, and corresponding Read Codes.

^f^ Depression: *ICD-10* codes F06.3, F32-F33, F34.1, F41.2, F44.8, F92.0, and corresponding Read Codes.

^g^ Bipolar disorder: *ICD-10* codes F30-31 and corresponding Read Codes.

^h^ Schizophrenia: *ICD-10* codes F06.2, F20, F23.0-F23.2, and corresponding Read Codes.

^i^ Antidepressant use: tricyclic and related antidepressant drugs, monoamine-oxidase inhibitors, selective serotonin reuptake inhibitors, and other antidepressant drugs.

Regression estimates from multiply imputed flexible parametric survival models are shown in Table 3. Log − log survival plots were used to check the proportional hazards assumption visually, and all models satisfied the assumption. In addition, the Figure displays estimates from the maximally adjusted multivariable models for both SARI and COVID-19 infection. All adjusted models showed that a severe outcome from COVID-19 and SARI was more likely in people with a preexisting diagnosis of a neuropsychiatric condition (COVID-19 smallest effect: anxiety diagnosis only, adjusted HR, 1.16; 99% CI, 1.12-1.20 and greatest effect: dementia diagnosis, HR, 2.85; 99% CI, 2.71-3.00; SARI smallest effect: anxiety diagnosis only, HR, 1.16; 99% CI, 1.13-1.18 and greatest effect: psychotic disorder diagnosis and treatment, HR, 2.56; 99% CI, 2.40-2.72). Effect estimates for all neuropsychiatric conditions and treatments were broadly similar for both outcomes, although the effect estimate for the association between severe COVID-19 and dementia appeared greater than for that of SARI, including an association between severe COVID-19 and dementia (COVID-19 infection: HR, 2.85; 99% CI, 2.71-3.00 vs SARI: HR, 2.13; 99% CI, 2.07-2.19) (Figure). Results from sensitivity analyses with COVID-19– and SARI-specific mortality as the outcome and restricting follow-up time in the contemporary cohort to the prevaccination period were similar to the main findings of the study (eTables 1, 2, and 3 in the Supplement).

**Figure.**
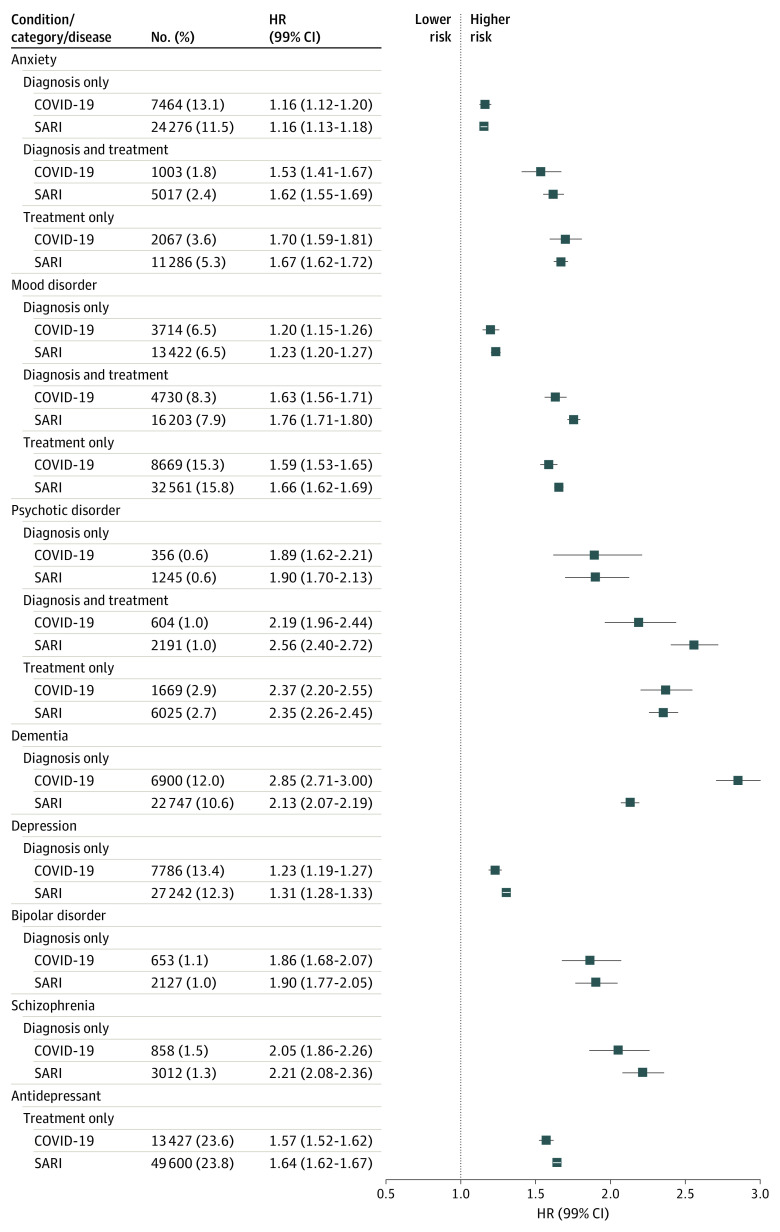
Forest Plot Showing Effect Estimates From Maximally Adjusted Survival Analyses of the Likelihood of Severe Acute Respiratory Infection (SARI) or COVID-19 Infection in People With a Diagnosed Neuropsychiatric Condition or Corresponding Pharmacological Treatment HR indicates hazard ratio.

## Discussion

Findings from the current cohort study suggest that people with neuropsychiatric conditions and/or associated pharmacological treatments have an associated increased risk of severe outcomes from COVID-19 infection and other SARIs. Furthermore, people with prescriptions for antidepressant medications had an associated increased risk of severe COVID-19 disease or SARI whether or not they had a corresponding diagnosis of a neuropsychiatric condition. Mainly, we found that people diagnosed with a neuropsychiatric condition or those prescribed corresponding psychotropic medications had a similarly associated increased risk of severe disease from COVID-19 and other SARIs, suggesting that these associations are not specific to COVID-19 infection. The magnitude of the effect estimate for risk of severe COVID-19 in people with dementia appeared greater than the corresponding value for SARI. This observation was made cautiously because the cohorts are temporally distinct, and other factors may have contributed to the difference, including the rapid and catastrophic spread of COVID-19 infection in nursing homes early in the pandemic in the UK.^22^

### Neuropsychiatric Conditions and COVID-19

Results of systematic reviews with meta-analysis of population-based cohort studies suggest that neuropsychiatric conditions (including anxiety, mood, psychotic, bipolar, personality, eating, and major depressive disorders; alcohol and substance abuse and misuse; and schizophrenia) are associated with an increased risk of COVID-19 mortality.^3,4,5^ Despite using slightly different definitions of severe illness, the direction of the effect estimates was consistent and the magnitude broadly similar with those of the current study, which used a composite outcome of hospitalization, ICU admission, and death. Together, the findings suggest that people with a diagnosis of a neuropsychiatric condition have an associated increased risk of severe outcome from COVID-19 infection. To the prior work, we add that the associated increased risks with COVID-19 are broadly similar to those seen in other SARIs.

### Neuropsychiatric Conditions and SARI

Cohort studies conducted before the COVID-19 pandemic have also reported an association between preexisting neuropsychiatric conditions and SARIs.^7,9^ A Danish population study^9^ conducted over 11 years found that people diagnosed with depression were at an associated increased risk of presenting with various respiratory infections. In addition, an English study^7^ of people from the Oxford region from 1999 to 2011 found that preexisting bipolar disorder, depression, and phobic anxiety conditions were each associated with a 2-fold increase in risk of pneumococcal lung infections. These findings are largely in line with those of the current study. Thus, 3 large observational cohort studies have found evidence suggesting that people with a preexisting neuropsychiatric condition are at an associated increased risk of severe outcome from SARIs.

### Psychotropic Medication

We found that people taking hypnotic, anxiolytic, antidepressant, or antipsychotic medication had an associated increased risk of more severe disease from both COVID-19 infection and SARI irrespective of whether they had a corresponding neuropsychiatric diagnosis, but findings from previous research are mixed. The current findings are consistent with evidence that SSRIs are associated with a higher risk of hospital mortality in patients admitted to ICU^13^ and with a systematic review and meta-analysis^10^ that found antipsychotic medication to be associated with increased risk of pneumonia. The authors of the systematic review, however, commented that there is a lack of randomized clinical trials in the area and that some observational studies failed to control for key confounders. In contrast, findings from a recent randomized clinical trial found that fluvoxamine, an SSRI, reduced the risk of hospitalization when given to symptomatic adult patients with an acute presentation consistent with COVID-19.^23^ It is interesting to compare the timing of drug administration. The current study and Ghassemi et al^13^ investigated SSRI use before tertiary care admission, whereas Reis et al^23^ investigated administration after the onset of symptoms consistent with COVID-19. Ultimately, the reason for the discrepancy is unknown, but the current study adds that the associated increased risks were not COVID-19 specific; rather, they were broadly similar to the risks in other severe respiratory conditions.

### Supplementary Analyses

Supplementary analyses showed that these associations were similar before the availability of vaccines for COVID-19 and when only the most severe outcome, mortality, was considered. This suggests that despite varying pressure on hospitals throughout the pandemic and the evolution of treatments and prophylactic measures, the association between neuropsychiatric conditions and severe COVID-19 infection and SARIs remained similar.

Although this study provided clear support for an association between neuropsychiatric conditions and more severe outcomes from respiratory infections, the observational design means it is not sufficient to demonstrate causality. We corrected for relevant demographic and clinical factors. However, it may be that neuropsychiatric illness occurs as part of a general picture of increased health disparity to the general population,^24^ which could increase the risk of opportunistic infection and lead to increased severity of disease from such an infection. Alternatively, some people with neuropsychiatric conditions may not have access to suitable clinical facilities or may delay clinical presentation until later in the disease stage, by which time the severity of their respiratory illness has increased.^24^ In either case, it seems clear that there is a generally elevated risk of severe acute respiratory illness associated with neuropsychiatric conditions and that this risk is similar with SARS-CoV-2 and other respiratory pathogens. This information is critical in determining the impact of neuropsychiatric illness on disease outcomes not only during the current pandemic but also into the future as COVID-19 becomes endemic.

### Strengths and Limitations

Strengths of our study include the use of large cohorts that are representative of the English general population and the use of multiple validated electronic health care data sets linked at the individual level to provide accurate ascertainment of exposures, outcomes, and other relevant confounders. These data sets minimize selection and recall bias because they use prospectively collected data. Furthermore, we used comparable respiratory infections to determine whether the reported association between neuropsychiatric conditions and severe COVID-19 was due to the SARS-CoV-2 pathogen specifically or a more general association with various pathogens that cause respiratory illness.

This study also has limitations. The use of routinely collected health care data means that outcomes and exposures are not formally adjudicated, as the database is dependent on coding by individual practitioners. There may also be recording bias due to disruption in patient attendance at general practices during the study period, particularly during the COVID-19 pandemic. We did, however, collect records from multiple linked databases including general primary care practice, hospital, and registry data, and this should serve to minimize potential bias from underreporting. Additionally, exclusion of people diagnosed during the study period may have introduced selection bias, although pilot analyses suggested that this was not the case, and we used a valid technique.^19^ Time-related heterogeneity can be masked by using HRs; however, the proportional hazards assumption held for all analyses, and sensitivity analysis varying follow-up time showed similar results to the main analyses. Finally, as with all observational studies, there is a risk of residual confounding, which we sought to minimize through adjustment for a range of confounders.

## Conclusions

The current cohort study builds on previous work in this field by investigating not only a range of neuropsychiatric conditions and psychotropic medications but by also comparing their associations with both severe COVID-19 infection and other SARIs. The adjusted results comparing severe outcomes from COVID-19 disease and other SARI largely seemed similar, suggesting that the associations were not disease specific. Although dementia was associated with a higher increased risk of severe outcome from COVID-19 than for SARI, well-documented impacts of the pandemic on specific care settings mean that this result should be interpreted with caution.

## Notes

Supplement. **eTable 1. **Showing Key Demographic Factors (Age, Sex, BMI), Outcomes and Exposures for COVID-19 in the Contemporary Cohort and SARI in the Prepandemic Cohort **eTable 2. **Results From Complete Analysis With SARI/COVID-19–Specific Mortality as the Outcome in the Prepandemic and Contemporary Cohorts, Respectively **eTable 3.** Results From Complete Case Analysis of the Contemporary Cohort Only Across the Whole Study Period and With the Study Period Restricted to Prior to Availability of the COVID-19 Vaccination (ie, Prior to 8 December 2020) in the Prevaccination Models, and Unrestricted (ie, Until 31 May 2021) in the Main Analysis

## References

[1] Dong E, Du H, Gardner L. An interactive web-based dashboard to track COVID-19 in real time. Lancet Infect Dis. 2020;20(5):533-534. doi:10.1016/S1473-3099(20)30120-1 32087114 PMC7159018

[2] Clift AK, Ranger TA, Patone M, . Neuropsychiatric ramifications of severe COVID-19 and other severe acute respiratory infections. JAMA Psychiatry. 2022;79(7):690-698. doi:10.1001/jamapsychiatry.2022.1067 35544272 PMC9096686

[3] Fond G, Nemani K, Etchecopar-Etchart D, . Association between mental health disorders and mortality among patients with COVID-19 in 7 countries: a systematic review and meta-analysis. JAMA Psychiatry. 2021;78(11):1208-1217. doi:10.1001/jamapsychiatry.2021.2274 34313711 PMC8317055

[4] Ceban F, Nogo D, Carvalho IP, . Association between mood disorders and risk of COVID-19 infection, hospitalization, and death: a systematic review and meta-analysis. JAMA Psychiatry. 2021;78(10):1079-1091. doi:10.1001/jamapsychiatry.2021.1818 34319365 PMC8319830

[5] Pardamean E, Roan W, Iskandar KTA, Prayangga R, Hariyanto TI. Mortality from coronavirus disease 2019 (COVID-19) in patients with schizophrenia: a systematic review, meta-analysis and meta-regression. Gen Hosp Psychiatry. 2022;75:61-67. doi:10.1016/j.genhosppsych.2022.01.010 35182908 PMC8813760

[6] Hassan L, Peek N, Lovell K, . Disparities in COVID-19 infection, hospitalisation and death in people with schizophrenia, bipolar disorder, and major depressive disorder: a cohort study of the UK Biobank. Mol Psychiatry. 2022;27(2):1248-1255. doi:10.1038/s41380-021-01344-2 34873324 PMC9054655

[7] Seminog OO, Goldacre MJ. Risk of pneumonia and pneumococcal disease in people with severe mental illness: English record linkage studies. Thorax. 2013;68(2):171-176. doi:10.1136/thoraxjnl-2012-202480 23242947

[8] Partti K, Vasankari T, Kanervisto M, . Lung function and respiratory diseases in people with psychosis: population-based study. Br J Psychiatry. 2015;207(1):37-45. doi:10.1192/bjp.bp.113.141937 25858177

[9] Andersson NW, Goodwin RD, Okkels N, . Depression and the risk of severe infections: prospective analyses on a nationwide representative sample. Int J Epidemiol. 2016;45(1):131-139. doi:10.1093/ije/dyv333 26708840

[10] Dzahini O, Singh N, Taylor D, Haddad PM. Antipsychotic drug use and pneumonia: systematic review and meta-analysis. J Psychopharmacol. 2018;32(11):1167-1181. doi:10.1177/0269881118795333 30334664

[11] Nosè M, Recla E, Trifirò G, Barbui C. Antipsychotic drug exposure and risk of pneumonia: a systematic review and meta-analysis of observational studies. Pharmacoepidemiol Drug Saf. 2015;24(8):812-820. doi:10.1002/pds.3804 26017021

[12] Chang CK, Chen PH, Pan CH, . Antipsychotic medications and the progression of upper respiratory infection to pneumonia in patients with schizophrenia. Schizophr Res. 2020;222:327-334. doi:10.1016/j.schres.2020.05.013 32507380

[13] Ghassemi M, Marshall J, Singh N, Stone DJ, Celi LA. Leveraging a critical care database: selective serotonin reuptake inhibitor use prior to ICU admission is associated with increased hospital mortality. Chest. 2014;145(4):745-752. https://journal.publications.chestnet.org/data/Journals/CHEST/929928/chest_145_4_745.pdf. doi:10.1378/chest.13-1722 24371841 PMC3971969

[14] Hennessy S, Bilker WB, Leonard CE, . Observed association between antidepressant use and pneumonia risk was confounded by comorbidity measures. J Clin Epidemiol. 2007;60(9):911-918. doi:10.1016/j.jclinepi.2006.11.022 17689807 PMC2042508

[15] University of Oxford. Investigating neuropsychological disease and treatments before and after severe COVID-19 disease. Accessed July 31, 2021. https://www.qresearch.org/media/1338/ox79-qresearch-icnarc-covid-19-psychological-outcomes.pdf

[16] von Elm E, Altman DG, Egger M, Pocock SJ, Gøtzsche PC, Vandenbroucke JP; STROBE Initiative. The strengthening the reporting of observational studies in epidemiology (STROBE) statement: Guidelines for reporting observational studies. Int J Surg. 2014;12(12):1495-1499. doi:10.1016/j.ijsu.2014.07.013 25046131

[17] National Institute for Health and Care Excellence. British National Formulary. Accessed August 31, 2021. https://bnf.nice.org.uk/

[18] QResearch. QCode group library. Accessed April 6, 2022. https://www.qresearch.org/data/qcode-group-library/

[19] Zhou Z, Rahme E, Abrahamowicz M, Pilote L. Survival bias associated with time-to-treatment initiation in drug effectiveness evaluation: a comparison of methods. Am J Epidemiol. 2005;162(10):1016-1023. doi:10.1093/aje/kwi307 16192344

[20] White IR, Royston P, Wood AM. Multiple imputation using chained equations: Issues and guidance for practice. Stat Med. 2011;30(4):377-399. doi:10.1002/sim.4067 21225900

[21] Rubin DB. Multiple Imputation for Nonresponse in Surveys. John Wiley & Sons Inc; 1989.

[22] Whatley E. Deaths involving COVID-19 in the care sector, England, and Wales: deaths registered between week ending 20 March 2020 and week ending 2 April 2021. Accessed January 8, 2022. https://www.ons.gov.uk/peoplepopulationandcommunity/birthsdeathsandmarriages/deaths/articles/deathsinvolvingcovid19inthecaresectorenglandandwales/deathsregisteredbetweenweekending20march2020andweekending2april2021

[23] Reis G, Augusto E, Carla D, . Effect of early treatment with fluvoxamine on risk of emergency care and hospitalisation among patients with COVID-19: the TOGETHER randomised, platform clinical trial. Lancet Glob Health. 2022;10(1):e42-e51. doi:10.1016/S2214-109X(21)00448-4 34717820 PMC8550952

[24] Firth J, Siddiqi N, Koyanagi A, . The Lancet Psychiatry Commission: a blueprint for protecting physical health in people with mental illness. Lancet Psychiatry. 2019;6(8):675-712. doi:10.1016/S2215-0366(19)30132-4 31324560

