# Supplementary material for: Preexisting Neuropsychiatric Conditions and Associated Risk of Severe COVID-19 Infection and Other Acute Respiratory Infections
Source: JAMA Psychiatry. 2022 Nov 9;80(1):57–65. doi: 10.1001/jamapsychiatry.2022.3614 (PMC9647578; doi:10.1001/jamapsychiatry.2022.3614)
Supplement: Supplement. — eTable 1. Showing Key Demographic Factors (Age, Sex, BMI), Outcomes and Exposures for COVID-19 in the Contemporary Cohort and SARI in the Prepandemic Cohort eTable 2. Results From Complete Analysis With SARI/COVID-19–Specific Mortality as the Outcome in the Prepandemic and Contemporary Cohorts, Respectively eTable 3. Results From Complete Case Analysis of the Contemporary Cohort Only Across the Whole Study Period and With the Study Period Restricted to Prior to Availability of the COVID-19 Vaccination (ie, Prior to 8 December 2020) in the Prevaccination Models, and Unrestricted (ie, Until 31 May 2021) in the Main Analysis [file jamapsychiatry-e223614-s001.pdf]

Supplementary Online Content

Ranger TA, Clift AK, Patone M, et al. Preexisting neuropsychiatric conditions and associated risk of severe COVID-19 infection and other acute respiratory infections. *JAMA Psychiatry*. Published online November 9, 2022. doi:10.1001/jamapsychiatry.2022.3614

eTable 1. Showing Key Demographic Factors (Age, Sex, BMI), Outcomes and Exposures for COVID-19 in the Contemporary Cohort and SARI in the Prepandemic Cohort

eTable 2. Results From Complete Analysis With SARI/COVID-19–Specific Mortality as the Outcome in the Prepandemic and Contemporary Cohorts, Respectively

eTable 3. Results From Complete Case Analysis of the Contemporary Cohort Only Across the Whole Study Period and With the Study Period Restricted to Prior to Availability of the COVID-19 Vaccination (ie, Prior to 8 December 2020) in the Prevaccination Models, and Unrestricted (ie, Until 31 May 2021) in the Main Analysis

This supplementary material has been provided by the authors to give readers additional information about their work.

© 2022 Ranger TA et al. *JAMA Psychiatry.*

eTable 1. Showing Key Demographic Factors (Age, Sex, BMI), Outcomes and Exposures for COVID-19 in the Contemporary Cohort and SARI in the Prepandemic Cohort

	Pre-pandemic cohort – SARI (2015/ 2020)		Contemporary cohort – COVID-19 (2020/ 2021)		
	Main analysis	Mortality analysis	Main analysis	Mortality analysis	Pre-vaccine analysis
Total	11,134,789	11,173,713	8,388,956	8,388,956	8,388,956
Age, median years (Q1-Q3)	42 (29- 58)	42 (29-58)	48 (34- 63)	48 (34-63)	48 (34-63)
Sex (% female)	5,644,525 (50.7)	5,665,688 (50.7)	4,181,764 (49.8)	4181764 (49.8)	4181764 (49.8)
BMI, median kg/m^2 (IQR)	26 (23- 30)	26 (23-30)	26 (23- 30)	26 (23-30)	26 (23-30)
BMI missing	1,773,555 (15.9)		1,212,590 (14.5)	1,212,453 (14.5)	1,225,889 (14.6)
First recorded outcome	223,569 (2.0)	-	58,203 (0.7)	-	30,996 (0.4)
Hospital	196,232 (1.8)	-	56,592 (0.7)	-	28,190 (0.3)
ICU	17,025 (0.2)	-	6,410 (0.1)	-	3,122 (0.0)
Death[1]	82,557 (0.7)	71,065 (0.6)	21,649 (0.3)	18,732 (0.2)	11,022 (0.1)
Anxiety[2]					
- Diagnosis only	999,125 (9.0)	1,003,942 (9.0)	1,013,565 (12.1)	1,013,565 (12.1)	1,013,565 (12.1)
- Diagnosis and treatment	62,194 (0.6)	63,221 (0.6)	50,899 (0.6)	50,899 (0.6)	50,899 (0.6)
- Treatment only	114,993 (1.0)	117,208 (1.0)	74,909 (0.9)	74,896 (0.9)	75,305 (0.9)
Mood disorder2					
- Diagnosis only	585,993 (5.3)	596,107 (5.3)	514,007 (6.1)	514,007 (6.1)	514,007 (6.1)
- Diagnosis and treatment	366,144 (3.3)	380,893 (3.4)	348,768 (4.2)	348,768 (4.2)	348,768 (4.2)
- Treatment only	603,914 (5.4)	621,406 (5.6)	576,955 (6.9)	576,941 (6.9)	577,535 (6.9)
Psychotic disorder2					
- Diagnosis only	30,262 (0.3)	30,668 (0.3)	23,194 (0.3)	23,194 (0.3)	23,194 (0.3)
- Diagnosis and treatment	34,519 (0.3)	35,172 (0.3)	26,866 (0.3)	26,866 (0.3)	26,866 (0.3)
- Treatment only	82,698 (0.7)	84,591 (0.8)	67,288 (0.8)	67,283 (0.8)	67,360 (0.8)
Dementia2	132,622 (1.2)	139,343 (1.2)	101,777 (1.2)	101,777 (1.2)	101,777 (1.2)
	Pre-pandemic		Contemporary cohort –		

[1] Counts of COVID-19/ SARI specific mortality change between the cohorts for the following reasons: in the pre-vaccination cohort the time period is restricted, so deaths occurring after 7 December 2020 are excluded; in the mortality specific cohorts some deaths occurred after an earlier outcome (i.e. hospital or ICU admission) but after study exit for another reason (e.g. deregistered from an EMIS practice), these are included in the main analysis but not the mortality specific analysis.

[2] Diagnoses recorded prior to baseline and/ or two prescriptions in the six months prior to baseline

© 2022 Ranger TA et al. *JAMA Psychiatry.*

	cohort – SARI (2015/ 2020)		COVID-19 (2020/ 2021)		
	Main analysis	Mortality analysis	Main analysis	Mortality analysis	Pre-vaccine analysis
Schizophrenia2	55,374 (0.5)	56,298 (0.5)	42,061 (0.5)	42,061 (0.5)	42,061 (0.5)
Depression2	921,948 (8.3)	928,102 (8.3)	825,732 (9.8)	825,732 (9.8)	825,732 (9.8)
Bipolar2	46,379 (0.4)	46,973 (0.4)	38,491 (0.5)	38,491 (0.5)	38,491 (0.5)
Anti-depressant treatment2	1,017,859 (9.1)	1028,950 (9.2)	928,341 (11.1)	928,341 (11.1)	928,341 (11.1)
Region of England					
- East Midlands	307,004 (2.8)	307,647 (2.8)	205,770 (2.5)	205,770 (2.5)	205,770 (2.5)
- East of England	419,157 (3.8)	420,401 (3.8)	325,733 (3.9)	325,733 (3.9)	325,733 (3.9)
- London	2,905,806 (26.1)	2,913,422 (26.1)	2,035,931 (24.3)	2,035,931 (24.3)	2,035,931 (24.3)
- North East	269,553 (2.4)	270,192 (2.4)	206,092 (2.5)	206,092 (2.5)	206,092 (2.5)
- North West	2,002,153 (18.0)	201,0074 (18.0)	1,578,863 (18.8)	1,578,863 (18.8)	157,8863 (18.8)
- South Central	1,415,670 (12.7)	1,420,650 (12.7)	1,089,524 (13.0)	1,089,524 (13.0)	1,089,524 (13.0)
- South East	1,166,717 (10.5)	1,172,856 (10.5)	915,109 (10.9)	915,109 (10.9)	915,109 (10.9)
- South West	1,131,089 (10.2)	1,135,160 (10.2)	859,409 (10.2)	859,409 (10.2)	859,409 (10.2)
- West Midlands	106,9962 (9.6)	1,074,615 (9.6)	845,864 (10.1)	845,864 (10.1)	845,864 (10.1)
- Yorkshire & Humber	44,7678 (4.0)	448,696 (4.0)	32,6661 (3.9)	326,661 (3.9)	326,661 (3.9)
Townsend quintile					
- 1 (Least deprived)	2,583,677 (23.2)	2,591,734 (23.2)	2,083,583 (24.8)	2,083,583 (24.8)	2,083,583 (24.8)
- 2	2,332,640 (20.9)	2,341,260 (21.0)	1,834,284 (21.9)	1,834,284 (21.9)	1,834,284 (21.9)
- 3	2,159,320 (19.4)	2,167,954 (19.4)	1,637,994 (19.5)	1,637,994 (19.5)	1,637,994 (19.5)
- 4	2,012,583 (18.1)	2,020,187 (18.1)	1,452,158 (17.3)	1,452,158 (17.3)	1,452,158 (17.3)
- 5 (Most deprived)	2,002,838 (18.0)	2,008,677 (18.0)	1,342,583 (16.0)	1,342,583 (16.0)	1,342,583 (16.0)
- Missing	43,731 (0.4)	43,901 (0.4)	38,354 (0.5)	38,354 (0.5)	38,354 (0.5)
Ethnicity					
- White	7,094,605 (63.7)	7,121,698 (63.7)	5,389,025 (64.2)	5,389,025 (64.2)	5,389,025 (64.2)
- Asian	986,264 (8.9)	988,457 (8.8)	735,644 (8.8)	735,644 (8.8)	735,644 (8.8)
- Black	381,244 (3.4)	382,201 (3.4)	285,126 (3.4)	285,126 (3.4)	285,126 (3.4)
	Pre-pandemic		Contemporary cohort –		

© 2022 Ranger TA et al. *JAMA Psychiatry.*

	cohort – SARI (2015/ 2020)		COVID-19 (2020/ 2021)		
	Main analysis	Mortality analysis	Main analysis	Mortality analysis	Pre-vaccine analysis
- Other	402,133 (3.6)	402,993 (3.6)	289,126 (3.4)	289,126 (3.4)	289,126 (3.4)
- Missing	2,270,543 (20.4)	2,278,364 (20.4)	1,690,035 (20.1)	1,690,035 (20.1)	1,690,035 (20.1)
Smoking status					
- non smoker	6,393,932 (57.4)	6,412,646 (57.4)	4,840,921 (57.7)	4,841,157 (57.7)	4,834,626 (57.6)
- ex smoker	2,325,940 (20.9)	2,342,255 (21.0)	1,827,288 (21.8)	1,827,338 (21.8)	1,823,075 (21.7)
- light smoker	1,506,588 (13.5)	1,509,433 (13.5)	1,050,847 (12.5)	1,050,746 (12.5)	1,061,123 (12.6)
- moderate smoker	290,564 (2.6)	290,777 (2.6)	229,416 (2.7)	229,508 (2.7)	227,010 (2.7)
- heavy smoker	134,483 (1.2)	134,645 (1.2)	106,090 (1.3)	105,835 (1.3)	104,593 (1.2)
- missing	483,282 (4.3)	2259834 (20.2)	334,394 (4.0)	334,372 (4.0)	338,529 (4.0)
Alcohol consumption					
- Non drinker	5,703,941 (51.2)	5,731,164 (51.3)	4,382,572 (52.2)	4,381,893 (52.2)	4,370,384 (52.1)
- Trivial <1u/day	1,631,593 (14.7)	1,636,947 (14.6)	1,249,772 (14.9)	1,250,171 (14.9)	1,251,581 (14.9)
- Light 1-2u/day	825,218 (7.4)	826,338 (7.4)	635,506 (7.6)	635,258 (7.6)	633,792 (7.6)
- Moderate 3-6u/day	614,568 (5.5)	615,752 (5.5)	487,122 (5.8)	488,018 (5.8)	488,930 (5.8)
- Heavy 7-9u/day	49,275 (0.4)	49,499 (0.4)	38,936 (0.5)	38,552 (0.5)	39,067 (0.5)
- Very Heavy >9u/day	53,900 (0.5)	54,179 (0.5)	35,720 (0.4)	35,831 (0.4)	36,038 (0.4)
- Missing	2,256,294 (20.3)	2,259,834 (20.2)	1,559,328 (18.6)	1,559,233 (18.6)	1,569,164 (18.7)
Comorbidities					
Neoplasms					
- GI cancers	88,171 (0.8)	90,167 (0.8)	59,124 (0.7)	59,197 (0.7)	57,278 (0.7)
- Urogenital cancers	134,940 (1.2)	136,968 (1.2)	106,473 (1.3)	106,517 (1.3)	104,871 (1.3)
- Gynaecological cancers	33,977 (0.3)	34,393 (0.3)	26,995 (0.3)	27,009 (0.3)	26,504 (0.3)
- Breast cancer	133,106 (1.2)	134,589 (1.2)	109,137 (1.3)	109,154 (1.3)	107,943 (1.3)
- Lung cancer	29,137 (0.3)	31,729 (0.3)	13,742 (0.2)	13,782 (0.2)	13,133 (0.2)
- Haematological cancers	63,490 (0.6)	65,387 (0.6)	50,349 (0.6)	50,415 (0.6)	49,161 (0.6)
Pulmonary					
	Pre-pandemic		Contemporary cohort –		

© 2022 Ranger TA et al. *JAMA Psychiatry.*

	cohort – SARI (2015/ 2020)		COVID-19 (2020/ 2021)		
	Main analysis	Mortality analysis	Main analysis	Mortality analysis	Pre-vaccine analysis
- COPD	279,977 (2.5)	292,026 (2.6)	209,515 (2.5)	209,607 (2.5)	208,477 (2.5)
- Asthma	1,473,736 (13.2)	1,483,329 (13.3)	1,147,463 (13.7)	1,147,527 (13.7)	1,144,133 (13.6)
- Bronchiectasis	55,855 (0.5)	59,185 (0.5)	47,767 (0.6)	47,817 (0.6)	47,095 (0.6)
- Rare pulmonary diseases	30,820 (0.3)	33,078 (0.3)	22,154 (0.3)	22,238 (0.3)	21,578 (0.3)
Circulatory					
- Coronary heart disease	436,822 (3.9)	445,907 (4.0)	333,273 (4.0)	333,577 (4.0)	328,502 (3.9)
- Hypertension	1,913,743 (17.2)	1,932,881 (17.3)	1,575,482 (18.8)	1,575,791 (18.8)	1,557,453 (18.6)
- Congestive cardiac failure	160,651 (1.4)	171,771 (1.5)	119,851 (1.4)	120,301 (1.4)	115,734 (1.4)
- Stroke	282,525 (2.5)	290,378 (2.6)	206,990 (2.5)	207,313 (2.5)	202,777 (2.4)
- Peripheral vascular dis.	98,505 (0.9)	100,994 (0.9)	69,596 (0.8)	69,660 (0.8)	68,440 (0.8)
- Venous thromboembolism	211,999 (1.9)	219,640 (2.0)	171,474 (2.0)	173,091 (2.1)	167,425 (2.0)
- Atrial fibrillation	308,170 (2.8)	321,726 (2.9)	237,653 (2.8)	238,401 (2.8)	231,861 (2.8)
- Sickle cell anaemia	8,699 (0.1)	8,955 (0.1)	6,976 (0.1)	6,979 (0.1)	6,928 (0.1)
Neurological					
- Learning impediments	177,637 (1.6)	179,404 (1.6)	137,143 (1.6)	137,166 (1.6)	136,165 (1.6)
- Severe head injury	58,509 (0.5)	58,793 (0.5)	46,947 (0.6)	46,947 (0.6)	46,837 (0.6)
- Epilepsy	151,385 (1.4)	153,385 (1.4)	116,631 (1.4)	116,671 (1.4)	116,085 (1.4)
- Multiple sclerosis	27,015 (0.2)	27,302 (0.2)	22,072 (0.3)	22,077 (0.3)	21,899 (0.3)
Skeletal					
- Fracture	471,836 (4.2)	480,490 (4.3)	355,081 (4.2)	355,367 (4.2)	351,468 (4.2)
- Osteoarthritis	1,200,356 (10.8)	121,3427 (10.9)	997,346 (11.9)	997,452 (11.9)	987,079 (11.8)
Other conditions					
- Rheumatoid diseases	98,820 (0.9)	100,375 (0.9)	81,073 (1.0)	81,091 (1.0)	80,232 (1.0)
- T1DM	64,244 (0.6)	64,900 (0.6)	48,433 (0.6)	48,464 (0.6)	48,129 (0.6)
- T2DM	753,013 (6.8)	763,600 (6.8)	623,174 (7.4)	623,699 (7.4)	612,203 (7.3)
- Hypothyroidism	478,928 (4.3)	483,721 (4.3)	386,816 (4.6)	386,876 (4.6)	383,777 (4.6)
- Renal complications	515,863 (4.6)	527,716 (4.7)	388,947 (4.6)	389,158 (4.6)	380,770 (4.5)
	Pre-pandemic		Contemporary cohort –		

© 2022 Ranger TA et al. *JAMA Psychiatry.*

	cohort – SARI (2015/ 2020)		COVID-19 (2020/ 2021)		
	Main analysis	Mortality analysis	Main analysis	Mortality analysis	Pre-vaccine analysis
- Chronic liver/ pancreatic diseases	88,689 (0.8)	90,583 (0.8)	82,501 (1.0)	82,692 (1.0)	77,870 (0.9)
- Bone marrow transplant	4,466 (0.0)	4,696 (0.0)	3,689 (0.0)	3,695 (0.0)	3,634 (0.0)
Medications					
- Steroids	815,596 (7.3)	839,571 (7.5)	289,510 (3.5)	290,941 (3.5)	253,004 (3.0)
- Statins	1,693,840 (15.2)	1,712,198 (15.3)	1,314,861 (15.7)	1,315,966 (15.7)	1,280,083 (15.3)
- NSAIDs	1,879,856 (16.9)	1,891,281 (16.9)	564,435 (6.7)	565,303 (6.7)	472,609 (5.6)
- Aspirin	690,868 (6.2)	701,826 (6.3)	384,181 (4.6)	384,943 (4.6)	369,468 (4.4)
- Oestrogen	216,352 (1.9)	217,384 (1.9)	146,110 (1.7)	146,176 (1.7)	136,314 (1.6)
- Progestogen	221,259 (2.0)	222,216 (2.0)	55,793 (0.7)	55,846 (0.7)	47,119 (0.6)
- HRT combined	99,658 (0.9)	100,145 (0.9)	47,564 (0.6)	47,587 (0.6)	42,628 (0.5)
- Anti-convulsants	673,395 (6.0)	688,130 (6.2)	373,428 (4.5)	374,316 (4.5)	348,495 (4.2)
- Bisphosphonates	255,609 (2.3)	263,670 (2.4)	134,537 (1.6)	134,906 (1.6)	126,786 (1.5)
- Leukotrienes	1,390,716 (12.5)	1,415,950 (12.7)	781,972 (9.3)	783,586 (9.3)	729,710 (8.7)
- ACE inhibitors	1,108,285 (10.0)	1,121,015 (10.0)	770,690 (9.2)	771,461 (9.2)	751,486 (9.0)
- Anti-coagulants	358,004 (3.2)	375,716 (3.4)	274,708 (3.3)	277,424 (3.3)	261,363 (3.1)
- Cytotoxins	33,888 (0.3)	34,837 (0.3)	18,641 (0.2)	18,700 (0.2)	17,660 (0.2)

© 2022 Ranger TA et al. *JAMA Psychiatry.*

eTable 2. Results From Complete Analysis With SARI/COVID-19–Specific Mortality as the Outcome in the Prepandemic and Contemporary Cohorts, Respectively

		SARI (pre-pandemic cohort)			COVID-19 (contemporary cohort)	
		Univariate model	Max. Adjusted model		Univariate model	Max. Adjusted model
	Cases	HR (99%CI)	HR (99%CI)	Cases	HR (99%CI)	HR (99%CI)
Anxiety						
No anxiety	217392 (80.0%)	1.00 (REF)	1.00 (REF)	14683 (80.2%)	1.00 (REF)	1.00 (REF)
Diagnosis only	32594 (12.0%)	1.39 (1.35, 1.42)	1.19 (1.17, 1.22)	2228 (12.2%)	1.07 (1.00, 1.13)	1.16 (1.08, 1.24)
Diagnosis and treatment	6748 (2.5%)	4.49 (4.32, 4.68)	1.64 (1.57, 1.71)	416 (2.3%)	4.02 (3.50, 4.61)	1.63 (1.40, 1.91)
Treatment only	15059 (5.5%)	5.83 (5.63, 6.03)	1.64 (1.59, 1.70)	987 (5.4%)	6.58 (5.98, 7.23)	1.79 (1.61, 1.99)
Mood disorders						
No mood disorder	178140 (67.8%)	1.00 (REF)	1.00 (REF)	12310 (67.4%)	1.00 (REF)	1.00 (REF)
Diagnosis only	18417 (7.0%)	1.48 (1.43, 1.53)	1.33 (1.29, 1.37)	997 (5.5%)	1.06 (0.97, 1.16)	1.09 (0.99, 1.21)
Diagnosis and treatment	22437 (8.5%)	2.85 (2.76, 2.95)	1.88 (1.83, 1.93)	1543 (8.5%)	2.40 (2.22, 2.59)	1.71 (1.56, 1.86)
Treatment only	43746 (16.6%)	3.42 (3.32, 3.52)	1.70 (1.66, 1.73)	3408 (18.7%)	3.22 (3.03, 3.43)	1.72 (1.61, 1.84)
Psychotic disorders						
No psychotic disorder	270380 (95.0%)	1.00 (REF)	1.00 (REF)	17258 (94.0%)	1.00 (REF)	1.00 (REF)
Diagnosis only	1981 (0.7%)	3.03 (2.84, 3.24)	2.32 (2.06, 2.61)	102 (0.6%)	2.22 (1.71, 2.90)	1.70 (1.24, 2.33)
Diagnosis and treatment	3255 (1.1%)	3.85 (3.64, 4.08)	2.91 (2.75, 3.08)	223 (1.2%)	4.01 (3.35, 4.81)	3.02 (2.47, 3.70)
Treatment only	8950 (3.1%)	4.97 (4.75, 5.19)	2.62 (2.50, 2.73)	773 (4.2%)	5.65 (5.07, 6.31)	3.08 (2.72, 3.48)
Other diagnoses						
Dementia	241251 (87.5%)	1.00 (REF)	1.00 (REF)	14010 (76.3%)	1.00 (REF)	1.00 (REF)
	34545 (12.5%)	19.64 (18.94, 20.37)	2.92 (2.81, 3.03)	4362 (23.7%)	30.22 (28.25, 32.34)	3.28 (3.04, 3.53)
Depression	250855 (87.1%)	1.00 (REF)	1.00 (REF)	16390 (87.5%)	1.00 (REF)	1.00 (REF)
	37309 (12.9%)	1.60 (1.55, 1.64)	1.35 (1.32, 1.38)	2331 (12.5%)	1.30 (1.22, 1.38)	1.18 (1.10, 1.27)
Bipolar disorder	289782 (98.9%)	1.00 (REF)	1.00 (REF)	18529 (99.0%)	1.00 (REF)	1.00 (REF)
	3121 (1.1%)	2.81 (2.67, 2.97)	2.07 (1.92, 2.24)	195 (1.0%)	2.32 (1.93, 2.81)	1.93 (1.56, 2.38)
Schizophrenia	288299 (98.4%)	1.00 (REF)	1.00 (REF)	18445 (98.5%)	1.00 (REF)	1.00 (REF)
	4587 (1.6%)	3.34 (3.18, 3.52)	2.56 (2.40, 2.73)	285 (1.5%)	3.16 (2.69, 3.71)	2.36 (1.96, 2.83)
Antidepressant use	200423 (74.8%)	1.00 (REF)	1.00 (REF)	13319 (72.9%)	1.00 (REF)	1.00 (REF)
	67647 (25.2%)	3.06 (2.98, 3.14)	1.69 (1.66, 1.72)	4956 (27.1%)	2.89 (2.74, 3.05)	1.70 (1.60, 1.80)

© 2022 Ranger TA et al. *JAMA Psychiatry.*

SARI – severe acute respiratory illness; Max. Adjusted model – maximally adjusted multivariable model; HR – Hazard Ratio; 99%CI – 99% Confidence Interval
Multivariable model adjusted for: age, sex, BMI, ethnicity, Townsend index of socio-economic deprivation, smoking and alcohol consumption, comorbidities, other medications

© 2022 Ranger TA et al. *JAMA Psychiatry.*

eTable 3. Results From Complete Case Analysis of the Contemporary Cohort Only Across the Whole Study Period and With the Study Period Restricted to Prior to Availability of the COVID-19 Vaccination (ie, Prior to 8 December 2020) in the Prevaccination Models, and Unrestricted (ie, Until 31 May 2021) in the Main Analysis

		COVID-19 (contemporary cohort) Main analysis - complete case			COVID-19 (contemporary cohort) Pre-vaccine analysis – complete case	
		Univariate model	Max. Adjusted model		Univariate model	Max. Adjusted model
	Cases	HR (99%CI)	HR (99%CI)	Cases	HR (99%CI)	HR (99%CI)
Anxiety						
No anxiety	46611 (81.6%)	1.00 (REF)	1.00 (REF)	24687 (80.8%)	1.00 (REF)	1.00 (REF)
Diagnosis only	7464 (13.1%)	1.13 (1.09, 1.17)	1.15 (1.11, 1.19)	4050 (13.3%)	1.16 (1.10, 1.21)	1.19 (1.13, 1.25)
Diagnosis and treatment	1003 (1.8%)	3.06 (2.81, 3.34)	1.51 (1.37, 1.66)	567 (1.9%)	3.26 (2.90, 3.67)	1.53 (1.34, 1.74)
Treatment only	2067 (3.6%)	4.36 (4.08, 4.67)	1.65 (1.54, 1.77)	1252 (4.1%)	4.94 (4.53, 5.39)	1.75 (1.60, 1.92)
Mood disorders						
No mood disorder	39701 (69.9%)	1.00 (REF)	1.00 (REF)	20827 (68.4%)	1.00 (REF)	1.00 (REF)
Diagnosis only	3714 (6.5%)	1.23 (1.17, 1.29)	1.17 (1.11, 1.24)	1999 (6.6%)	1.27 (1.19, 1.36)	1.22 (1.14, 1.31)
Diagnosis and treatment	4730 (8.3%)	2.28 (2.18, 2.39)	1.56 (1.49, 1.64)	2612 (8.6%)	2.43 (2.28, 2.59)	1.68 (1.57, 1.79)
Treatment only	8669 (15.3%)	2.55 (2.45, 2.65)	1.51 (1.45, 1.57)	5023 (16.5%)	2.83 (2.69, 2.99)	1.64 (1.55, 1.74)
Psychotic disorders						
No psychotic disorder	55033 (95.4%)	1.00 (REF)	1.00 (REF)	29164 (94.9%)	1.00 (REF)	1.00 (REF)
Diagnosis only	356 (0.6%)	2.45 (2.10, 2.86)	1.64 (1.37, 1.96)	226 (0.7%)	2.89 (2.40, 3.46)	1.89 (1.53, 2.34)
Diagnosis and treatment	604 (1.0%)	3.43 (3.07, 3.82)	2.08 (1.85, 2.35)	356 (1.2%)	3.79 (3.28, 4.38)	2.37 (2.03, 2.77)
Treatment only	1669 (2.9%)	3.85 (3.56, 4.15)	2.22 (2.04, 2.42)	984 (3.2%)	4.24 (3.85, 4.69)	2.33 (2.09, 2.60)
Other diagnoses						
Dementia	50621 (88.0%)	1.00 (REF)	1.00 (REF)	26199 (85.4%)	1.00 (REF)	1.00 (REF)
	6900 (12.0%)	13.45 (12.78, 14.17)	2.71 (2.56, 2.86)	4496 (14.6%)	15.82 (14.77, 16.93)	2.82 (2.61, 3.04)
Depression	50370 (86.6%)	1.00 (REF)	1.00 (REF)	26766 (86.4%)	1.00 (REF)	1.00 (REF)
	7786 (13.4%)	3.08 (2.79, 3.40)	1.20 (1.15, 1.25)	4210 (13.6%)	1.44 (1.37, 1.51)	1.24 (1.17, 1.31)
Bipolar disorder	57514 (98.9%)	1.00 (REF)	1.00 (REF)	30596 (98.7%)	1.00 (REF)	1.00 (REF)
	653 (1.1%)	1.41 (1.36, 1.46)	1.80 (1.60, 2.02)	389 (1.3%)	2.81 (2.45, 3.22)	1.95 (1.67, 2.27)
Schizophrenia	57331 (98.5%)	1.00 (REF)	1.00 (REF)	30472 (98.3%)	1.00 (REF)	1.00 (REF)
	858 (1.5%)	2.52 (2.26, 2.81)	1.89 (1.70, 2.11)	515 (1.7%)	3.44 (3.03, 3.90)	2.12 (1.84, 2.44)
Antidepressant use	43469 (76.4%)	1.00 (REF)	1.00 (REF)	22849 (74.9%)	1.00 (REF)	1.00 (REF)
	13427 (23.6%)	2.41 (2.33, 2.49)	1.50 (1.45, 1.55)	7647 (25.1%)	2.63 (2.52, 2.75)	1.62 (1.55, 1.70)

© 2022 Ranger TA et al. *JAMA Psychiatry.*

Max. Adjusted model – maximally adjusted multivariable model; HR – Hazard Ratio; 99%CI – 99% Confidence Interval
Multivariable model adjusted for: age, sex, BMI, ethnicity, Townsend index of socio-economic deprivation, smoking and alcohol consumption, comorbidities, other medications

© 2022 Ranger TA et al. *JAMA Psychiatry.*
